# Views on Aging and Well-Being in the Covid Crisis – A Longitudinal Study in Luxembourg

**DOI:** 10.1093/geroni/igaa057.3513

**Published:** 2020-12-16

**Authors:** Anna Kornadt, Martine Hoffmann, Elke Murdock, Josepha Nell, Isabelle Albert

**Affiliations:** 1 University of Luxembourg, Esch-sur-Alzette, Diekirch, Luxembourg; 2 RBS Center fier Altersfroen, Itzig, Luxembourg; 3 University of Luxembourg, Esch-sur-Alzette, Luxembourg

## Abstract

During the Covid-Crisis, stereotypes of older adults as helpless and vulnerable were spread, and intergenerational conflict was stirred more or less openly. We thus focused on perceived ageism during the crisis and its effects on well-being and health of older adults. Since views on aging are multifaceted and can be both, risk and resource for individual development, we assessed people’s self-perceptions of aging (SPA) as social loss, continued growth and physical decline and subjective age (SA). We hypothesized that people with SPA of social loss and physical decline would be more susceptible to negative effects of perceived ageism, whereas those with SPA of continued growth and younger SA would be less affected. NT1 = 611 community-dwelling adults aged 60 – 98 (Mage = 69.92 years) were recruited in June 2020 online and via phone in Luxembourg. In September 2020, participants will be contacted again for a follow-up. Analyses with cross-sectional data show that participants who felt more discriminated reported lower life satisfaction after the onset of the crisis (r = -.35) and worse subjective health (r = -.14). SPA of social loss and higher SA increased the negative effect of ageism on well-being (beta = -.57) and subjective health (beta = -.53), respectively. Our results point to mid- and long-term consequences of age discriminatory and stereotype-based crisis communication for the well-being of older adults and the importance of individual SPA in critical situations.

